# An Antibiotic-Loaded Hydrogel Demonstrates Efficacy as Prophylaxis and Treatment in a Large Animal Model of Orthopaedic Device-Related Infection

**DOI:** 10.3389/fcimb.2022.826392

**Published:** 2022-04-27

**Authors:** Willemijn Boot, Andrew Lewis Foster, Olivier Guillaume, David Eglin, Tanja Schmid, Matteo D’Este, Stephan Zeiter, Robert Geoff Richards, Thomas Fintan Moriarty

**Affiliations:** ^1^AO Research Institute Davos, Davos, Switzerland; ^2^Queensland University of Technology Centre for Biomedical Technologies, Brisbane, QLD, Australia; ^3^Jamieson Trauma Institute, Metro North Hospital and Health Service, Brisbane, QLD, Australia; ^4^Department of Orthopaedic Surgery, Redcliffe Hospital, Queensland Health, Brisbane, QLD, Australia

**Keywords:** orthopaedic device related infection, local antibiotics, hydrogel, fracture-related infection, peri-prosthetic joint infection, preclinical

## Abstract

Local antibiotic therapy is increasingly being recognised for its role in preventing and treating orthopaedic device-related infection (ODRI). A bioresorbable, injectable gentamicin-loaded hydrogel has been developed to deliver local antibiotics at the time of surgery with potential for both prevention and treatment of ODRI. In a prophylaxis model, the antibiotic hydrogel was compared with systemic perioperative antibiotic prophylaxis alone in twelve sheep (six per group) at the time of intramedullary (IM) nail insertion to the tibia, which was inoculated with methicillin-sensitive *Staphylococcus aureus* (MSSA). In a treatment model of single-stage revision surgery, adjunctive antibiotic-loaded hydrogel was compared with systemic antibiotics alone in a single stage revision of MSSA infection associated with a tibia intramedullary nail in eleven sheep (five/six per group). The primary endpoint was quantitative microbiological results of soft tissue, bone and sonicate fluid from explanted hardware at the time of euthanasia. At euthanasia, the control sheep that received no local antibiotics in the prophylaxis model were all culture-positive (median 1x10^8^, range 7x10^6^-3x10^8^ colony forming units, CFU) while only two of six sheep receiving local gentamicin had any culture positive biopsies (median 1x10^1^, range 0 - 1x10^5^ CFU). For the treatment model, sheep receiving only systemic antibiotics were all culture-positive (median 8x10^5^, range 2x10^3^- 9x10^6^ CFU) while only two of six sheep treated with gentamicin-loaded hydrogel had any culture positive biopsies (median 3x10^2^, range 0 - 7x10^4^ CFU). Local gentamicin concentrations measured in extracellular fluid in the tibial canal show a burst release of gentamicin from the hydrogel. Serum gentamicin concentrations peaked in both models at one day post application and were below detection limit thereafter. This study has demonstrated the effective use of a locally delivered antibiotic hydrogel for both the prevention and treatment of ODRI that is superior to that of systemic antibiotics alone. Future studies will endeavour to translate from preclinical to clinical research trials.

## Introduction

Orthopaedic device related infection (ODRI) is a devastating complication following either elective or trauma surgery. Managing ODRI requires a multidisciplinary team due to the complexity of these cases (Anderson et al., 2019), often requiring multiple additional surgical procedures and a long duration of antibiotic therapy (Foster et al., 2020; Foster et al., 2020). *Staphylococcus aureus* is the most common infecting organism and its ability to form a biofilm and evade host immune defences largely account for the challenge in preventing and treating these infections from both a microbiological and surgical perspective (Arciola et al., 2005; Arciola et al., 2018). Despite best current practice, treatment outcomes remain suboptimal with those treated for an ODRI at one-year follow up reporting lower physical and mental health scores, less likely to return to work or retain their independence, increased number of re-hospitalisations and higher mortality rate compared to uninfected patients (Czaja et al., 2009). The health economic burden of treatment is immense, estimated to be 1.62 billion US dollars for hospital related costs in the United States alone in 2020 (Kurtz S et al., 2012), which does not include the lost productivity secondary to the aforementioned poor patient-related outcomes.

In addition to surgery, the medical management of ODRI largely consists of systemically delivered antibiotic therapy. In the context of prevention, perioperative antibiotic prophylaxis with a first-generation cephalosporin is administered within 1 hour prior to skin incision (Classen et al., 1992). In a treatment context, management includes empirical antibiotics followed by targeted intravenous antibiotics, followed by a course of oral agents for up to 3 months of treatment duration in total (Osmon et al., 2013). As an adjunct to systemic therapy, local antibiotic therapy is increasingly being recognised as a method to improve the prevention and treatment of ODRI (Kaur et al., 2014), while minimising the complications associated with systemic exposure (Metsemakers et al., 2019). Polymethyl methacrylate (PMMA) bone cement remains the most often used delivery vehicle for local antibiotics (Peel, 2019). This is used as prophylaxis for fracture-related infection (FRI) in open fractures when applied as beads, or in primary cemented arthroplasty (Morgenstern et al., 2018). It may also be used for treatment as a spacer in the surgical management of peri-prosthetic joint infection (PJI) (Abouljoud et al., 2019). Despite supporting treatment with high local concentrations of antimicrobials, antibiotic loaded bone cement (ALBC) has several limitations, including pharmacokinetic release, antibiotic compatibility, and the lack of degradation (Foster et al., 2020). The latter limits the practical use of ALBC in the prevention of FRI and single stage revision surgeries, where the use of PMMA often necessities further surgery for removal.

Hydrogels have been developed as a bioresorbable local delivery vehicle for antibiotics that can overcome many limitations of ALBC (Foster et al., 2020). In a small animal (rabbit) model of ODRI a gentamicin-loaded hydrogel has demonstrated efficacy in preventing methicillin-sensitive *S. aureus* (MSSA) infection (Ter Boo et al., 2016). Furthermore, in the same rabbit model, it has been demonstrated that there was no significant negative impact to fracture healing secondary to antibiotic hydrogel application (Boo et al., 2018). This hydrogel combined with gentamicin and vancomycin demonstrated efficacy against methicillin-resistant *S. aureus* (MRSA) ODRI in a large animal (sheep) model when used in both two-stage (Boot et al., 2021) and single-stage revision protocols (Foster et al., 2020). In these models the *in vivo* pharmacokinetic release of antibiotic hydrogel was found to measure 10-100 times the local concentration of that released by ALBC, while systemic levels were not significantly elevated nor were liver or renal biochemical markers.

In the process of developing and evaluating this antibiotic-loaded hydrogel for numerous surgical and microbiological indications, a research gap currently exists in the efficacy for prevention in a large animal model, and in the treatment of established MSSA infection. Our primary aim was to demonstrate the efficacy of a gentamicin-loaded hydrogel in prevention and treatment of a MSSA ODRI in sheep. The secondary aim was to evaluate local and systemic pharmacokinetics of systemically and locally applied antibiotics.

## Materials and Methods

### General Information

The ethical committee of the canton of Grisons in Switzerland approved this study (approval number 26/2015), which adheres to the ARRIVE guidelines for preclinical animal models (see supplemental material). A total of 27 skeletally mature (2 to 4 years old) female Swiss Alpine sheep, with an average weight of 69 kg (+/- 7.8), were enrolled in this study. Prior to inclusion, the sheep underwent a physical examination including a radiographical screening of the tibia to ensure that it could accommodate an intramedullary (IM) nail. The sheep were housed in groups for at least 2 weeks before surgery for acclimatisation. The sheep got daily cycles of 12 hours dark and light and were fed twice per day with hay, a mineral lich, and hand-fed grain to gain familiarity with the animal caretakers.

### Animal Model and Study Design

The animal models used in the present study are based on an earlier established device-related bone infection in sheep (Moriarty et al., 2017). All the animals received an intramedullary nail into the tibia during the primary surgery, while an infection was induced during the same surgery by inoculating the intramedullary canal of the tibia with a clinical methicillin-sensitive *S. aureus* (MSSA). During allocation to control or treatment groups, sheep were pseudo‐randomized by a research assistant during the operation. Complete randomization could not be completed due to the differing surgical equipment. On each surgical day, half of the sheep would be randomly allocated to each group.

#### Prophylaxis Model

For the prophylaxis model, all sheep received one intravenous dose of first-generation cephalosporin preoperatively. During surgery, bacteria were introduced by injecting bacteria through the inoculation hole and *via* placement of a piece of collagen fleece containing bacteria into the same hole. Subsequently, gentamicin-loaded hydrogel was applied to the intramedullary canal prior to the placement of the nail. The control group received no hydrogel nor local antibiotics. After a follow-up of 21 days, animals were euthanised and the operated limb harvested for bacteriology. Further details of group, surgical and medical treatments given below.

#### Treatment Model (Single Stage Revision)

For the single-stage treatment model, after establishing a chronic ODRI over 8 weeks, revision was performed to remove the hardware, debride the intramedullary canal, and place a new intramedullary nail. A washout period of two weeks was included after the systemic antibiotic treatment to reduce the risk of false negative culture results due to residual antibiotics at euthanasia. Antibiotic gel treated sheep were compared with a control-group, where the sheep received systemic antibiotic therapy but no local antibiotic treatment. Further details of group, surgical and medical treatments given in **Table 1**.

**Table 1:** Groups and treatment overview.

	Prophylaxis		One-stage treatment	
	Control	Hydrogel	Control	Hydrogel
Number of animals	6	6	5	6
Systemic perioperative antibiotic prophylaxis administered	Yes	Yes	Yes	Yes
Gentamicin-loaded hydrogel administered	No	During primary surgery	No	During revision surgery

### Bacteria and Inoculum Preparation

The MSSA strain used in the study was ATCC 25923, a human clinical isolate previously used in the earlier established device-related bone infection in sheep (Moriarty et al., 2017). To prepare the inoculum, bacteria were taken from a frozen glycerol stock, streaked out on tryptic soy agar plates (TSA) and incubated 16-18 hours. A single colony was picked, resuspended in 25 mL of tryptic soy broth (TSB) and incubated at 37°C for 14-16 hours under agitation (100 RPM). This bacterial suspension was washed twice with phosphate buffered saline (PBS) (centrifuged at 2500 RPM for 10 minutes). The bacterial suspension in PBS was sonicated for 3 minutes (Bandelin Sonorex Super 10P, Bandelin, Berlin, Germany) and vortexed for 30 s. The optical density of the bacterial suspension was adjusted at 600 nm to a range between 1.1 and 1.2 (MultiskanTM GO spectrophotometer, Thermo Scientific, Zürich, Switzerland). From this stock, 20 µL (approximately 2 × 10^7^ colony forming units (CFU)) was added to a collagen fleece (TissuFleece E, Baxter AG, Volketswil, Germany). Bacteria were introduced by injecting 200 µl of PBS with bacteria through the inoculation hole and *via* placement of the piece of collagen fleece containing bacteria through the same hole. Quantitative culture was performed on the stock solution after each inoculation by plating a serial 10-fold dilution on TSA plates to assess the total count of bacteria administered to each animal.

### Preparation of Gentamicin-Loaded Hydrogel

The poly(N-isopropylacrylamide) grafted hyaluronic acid hydrogel was prepared as previously described (D'Este et al., 2012; Ter Boo et al., 2016) by functionalizing the hyaluronic acid carboxy groups with amino-terminated poly(N-isopropylacrylamide) of Mw 28 kDa *via* amide formation. The lyophilized polymer was sterilized with cold ethylene oxide and degassed over a 5-day period. The hydrogel was reconstituted overnight at 4°C to a concentration of 11% in PBS with 1% gentamicin sulphate. Prior to insertion of the nail at primary surgery (prophylaxis model) or a new nail during revision surgery (one-stage model), 20 mL of antibiotic-loaded hydrogel was inserted into the intramedullary canal using a syringe attached to a soft plastic cannula (total of 200 mg Gentamicin per sheep),(Rauclair-E 4/1,5; Rehau Vertriebs AG, Switzerland).

### Primary Surgery

A detailed description of the procedure was published previously (Moriarty et al., 2017). Briefly, after induction of general anaesthesia, the left tibia and knee joint were prepared for aseptic surgery. A medial para-patellar approach was used to gain access to the tibial plateau. An entry point for the nail was created cranial to the stifle joint. A 190 mm long, 7.5 mm diameter UHN Depuy Synthes Nail (Ref.: 462.719) and a 3.9 mm locking bolt (one-stage model only) were placed. The bacterial inoculum was delivered on a collagen sponge through a 4.3 mm unicortical mid-diaphyseal drill hole through the medial cortex. All tissues were closed in layers with absorbable suture material.

### Revision Surgery (One-Stage Model Only)

Eight weeks following the index procedure, the sheep in the one-stage model underwent a single stage revision surgery. The same anaesthesia protocol was used as described previously (Moriarty et al., 2017). A medial parapatellar approach was used to extract the nail, and a stab incision was performed to remove the locking bolt, followed by removal of the nail. A simple debridement of the intramedullary canal was performed using a femoral canal brush (Smith and Nephew AG, London, UK) by inserting it through the nail entry point and turning it 90 degrees before removing the brush. Closure was performed as described for the initial surgery.

### Systemic Antibiotic Therapy (All Animals)

All animals received a systemic dose of cefazolin preoperatively (2.2 mg/kg) in both the prophylaxis and treatment studies.

Systemic antibiotic therapy was only required in the one-stage treatment study. Treatment began the day after revision surgery. Amoxicillin clavulanic acid was administered subcutaneously once per day as Synulox^®^ suspension (7.0 mg amoxicillin, 1.5 mg clavulanic acid/kg; Zoetis Schweiz GmbH, Zurich, Switzerland). Trimethoprim sulphadoxine was administered subcutaneously (s.c.) once per day as 24% Borgal^®^ suspension (12 mg sulphadoxine + 2.4 mg trimethoprim (3 mL/50 kg body weight); MSD Animal Health GmbH, Luzern, Switzerland).

### Radiographs

A high-resolution contact radiograph was taken post-mortem, using high-resolution technical film (D4 Structurix DW ETE, Agfa^®^, Belgium) and a cabinet X-ray system (Model No. 43855A, Faxitron X-Ray Corporation^®^, USA) at 42 kV for 5 min, with a 0.5 mm aluminium filter.

### Extracellular Fluid and Serum Sampling

All animals that received the hydrogel, also received an ultrafiltration probe to collect extracellular fluid (BASI Bioanalytical Systems, West Lafayette, MF-7028). The probe was inserted into the medullary cavity through the nail entry point. The end of the probe was pulled out of the medullary canal through the drill hole of the locking bolt. With the introducer needle (BASI Bioanalytical Systems, West Lafayette, MR-5313), the tissue was tunnelled for the probe to exit the skin on the lateral side of the leg, where it was fixed with stiches. The limb remained bandaged for the whole period the probe was placed, and bandages were changed regularly. The vials containing the ultrafiltration fluid were changed every morning and afternoon and stored at -20°C. For serum sampling, blood was taken preoperatively, and 2h, 3d, 1w and 2w postoperatively (prophylaxis model), and preoperatively, daily for 7 days after revision surgery and weekly thereafter (1-stage model). Blood was centrifuged (10 minutes, 2500 RPM) and serum was collected and stored at -20°C. Extracellular fluid and serum samples were sent to Lausanne University Hospital for analysis of gentamicin, and amoxicillin (one-stage model only).

### Animal Welfare and Observation, Euthanasia

Post-operative analgesia protocol after all surgeries consisted of subcutaneous carprofen (4 mg kg-1; Carprodolor; Virbac Switzerland AG) for 5 days, intramuscular buprenorphine (0,6 mg, Bupaq, Streuli Pharma AG), and topical fentanyl (2 µg/h Fentanyl-Mepha Matrixpflaster, Mepha Pharma AG, Basel) for 3 days. This regimen was provided as-required thereafter. The sheep were routinely checked by a veterinarian or an experienced animal caretaker for general behaviour, weight bearing on the operated leg, body temperature, respiration, appetite and defecation. A customized cumulative scoring system was used to assess the burden on the animals and reaching a predefined score of >7 for 3 days results in early euthanasia. Euthanasia was performed with an intravenous overdose of pentobarbital (Esconarkon^®^, Streuli Pharma AG).

### Quantitative Bacteriology at Revision Surgery and Euthanasia

At revision surgery (one-stage model only), the hardware (nail and interlocking bolt) and brush used for debridement were collected for quantitative bacteriology. The nail and brush were placed in 60 mL of sterile PBS and agitated 10 times. The bolt was placed in 20 mL of sterile PBS and vortexed for 30 s. Subsequently, the samples were sonicated for 5 minutes and agitated and vortexed again.

Before starting dissection of the limb after euthanasia, high-resolution contact radiographs were made as described above. The leg was then carefully dissected and superficial tissues from the distal and proximal interlocking bolt holes and inoculation point were collected. The samples were weighed and homogenised in 20 mL PBS. Cortical bone biopsies from the distal and proximal interlocking bolt holes and the inoculation point were collected using a Partsch spoon. The samples were weighed and homogenised in 20 mL PBS. Bone marrow was collected and weighed and homogenised in 20 mL PBS (Omni-TH hand-held homogeniser with sterile Omni-tip plastic probes, LabForce AG, Switzerland). All samples were sonicated for 5 minutes. The nail and bolt were treated as described above. All samples were 10-fold serially diluted in sterile PBS and 200 μL of each dilution was spread onto blood agar plates. The plates were incubated at 37°C and colonies were counted after 24 and 48 h. Bacteria from brush or bone marrow from each animal was assessed by a *S. aureus*-specific latex agglutination test (Staphaurex Plus, Remel, Basel, Switzerland).

### Statistical Analyses

The group size was calculated to enable detection of a 70% reduction in infection rate with 80% power and 5% significance level. The primary outcome parameter was CFU compared between the test and control group with a two-tailed unpaired T-test using Log transformed CFU data, after normality testing, using GraphPad Prism. P values < 0.05 were considered statistically significant.

## Results

### Animal Welfare

One animal from the control group in the treatment model was replaced as it was euthanised due to an unrelated illness, and one animal from the same group showed a mixed infection at euthanasia and was declared as contaminated. This animal was excluded from the study but not replaced, therefore all outcome parameters from this control group are based on the result of 5 animals instead of 6. Two animals from the gentamicin-loaded hydrogel group (same model) showed severe symptoms from the infection before revision surgery took place and before any antibiotics were administered. The animals needed to be euthanised and were replaced.

### Prevention of ODRI

#### Bacterial Inoculum

The bacterial inoculum that was administered to each sheep was within the range of 8.00x10^6^ – 2.01x10^7^ CFUs per animal.

#### Bacteriology

**Figure 1:**
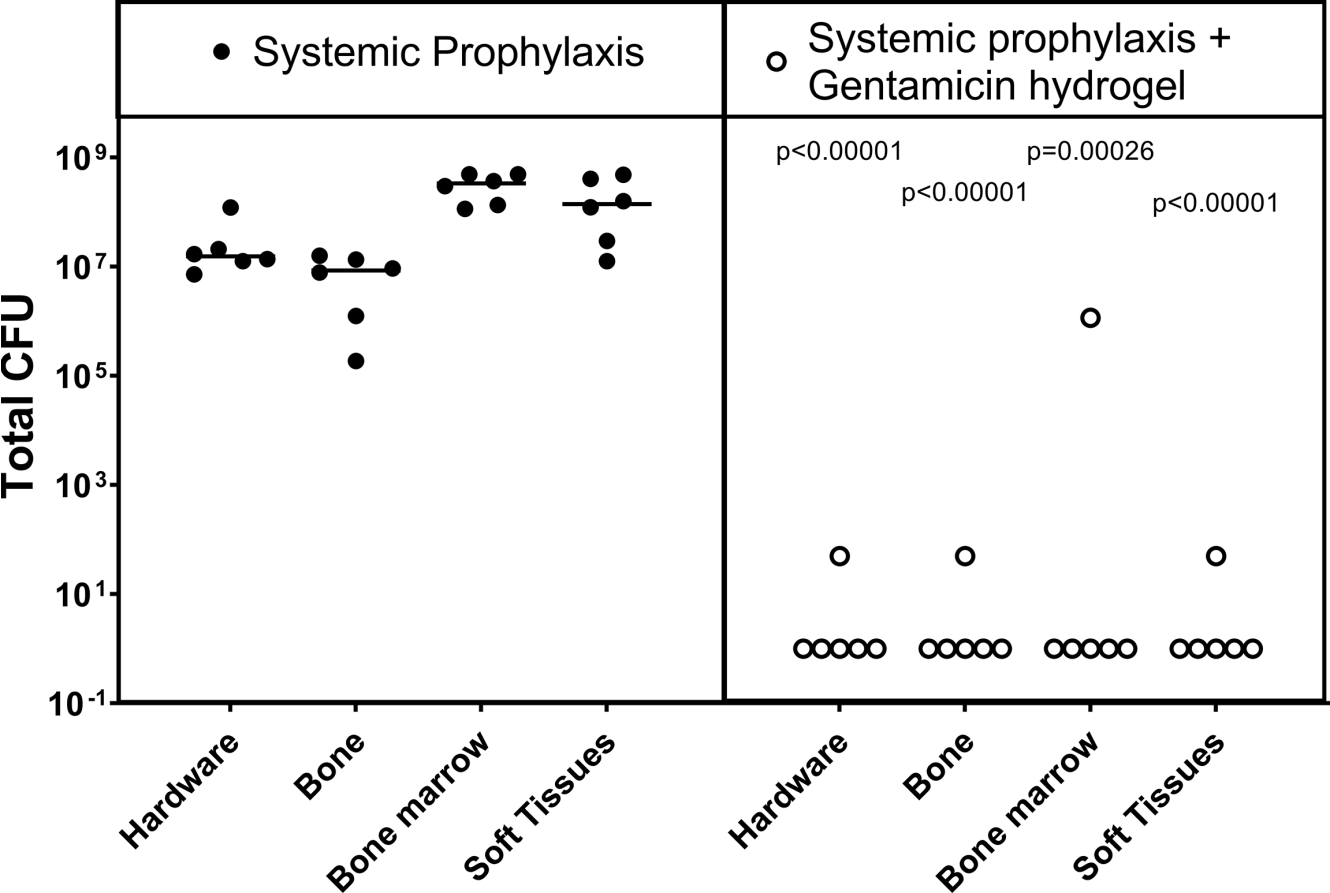
Bacteriology at euthanasia in the prophylaxis model. CFU counts were obtained from individually collected tissue samples taken postmortem and individually processed. Gentamicin hydrogel was applied immediately before wound closure. Statistical comparisons are per location for each group, n= 6 per group. Lines represent median values, statistical comparison by two-sided T-test of Log transformed data.

At euthanasia, the sheep in the control group were all culture positive (**Figure 1**), indicating that systemic prophylaxis alone was not sufficient to prevent an ODRI in this model (median 1x10^8^, range 7x10^6^ - 3x10^8^ CFU). Application of local gentamicin prevented orthopaedic-device related infection successfully in 4 sheep. One sheep had a culture-positive bone marrow sample, one sheep had a positive soft tissue sample (median 1x10^1^, range 0 - 1x10^5^ CFU).

### Treatment of ODRI

#### Bacterial Inoculum

The average number of CFUs administered to the sheep in the one-stage model was 1.17x10^7^ ± 2.62x10^6^ for the control group and 1.30x10^7^ ± 3.19x10^6^ for the sheep receiving hydrogel.

#### Bacteriology

**Figure 2:**
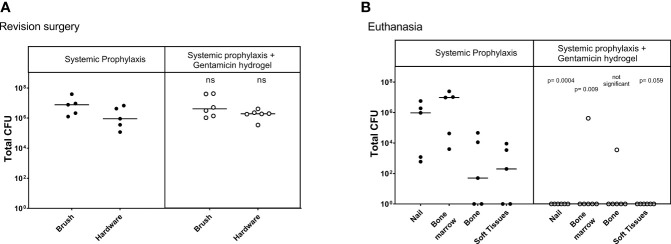
Bacteriology at revision (Left) and euthanasia (Right) in the one-stage treatment model. CFU counts were obtained from individually collected tissue samples during revision, where biopsies and hardware were removed, and at euthanasia, where the entire limb was dissected. Statistical comparisons are per location for each group. Lines represent median values, statistical comparison by two-sided T-test of Log transformed data.

At revision surgery, all 11 sheep were found to be infected with no statistical differences between both groups (**Figure 2**). All bacteria cultured from the brush and bone marrow samples were found to be *S. aureus* by latex agglutination tests.

At euthanasia, the sheep in the systemic antibiotic-only group were all culture positive (**Figure 2**), confirming that debridement and a one-stage exchange procedure including systemic antibiotic therapy did not treat the infection in this model (median 8x10^5^, range 2x10^3^ - 9x10^6^ CFU). The group treated with gentamicin-loaded hydrogel had a significant decrease of total CFU (sum of all biopsies) cultured at euthanasia (P<0.001). One animal in the treatment group had a culture positive bone marrow sample, and one animal in this group had a positive bone sample. The 4 other animals in this group were culture-negative at euthanasia (overall median 3x10^2^, range 0- 7x10^4^ CFU).

### Serum and Extracellular Fluid Antibiotic Concentrations

**Figure 3:**
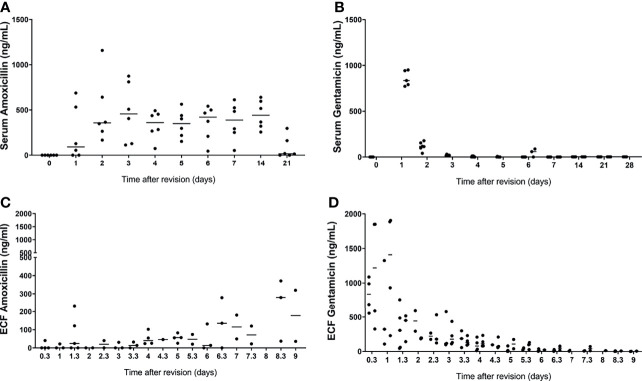
Serum and extracellular fluid amoxicillin (**A, C**, respectively) and gentamicin (**B, D**, respectively) concentrations from sheep that received the gentamicin-loaded hydrogel. Extracellular fluid was collected by ultrafiltration from the intramedullary canal of the tibia in the morning and afternoon, after application of the gentamicin-loaded hydrogel in both models. Serum was collected daily for 7 days and weekly thereafter after application of the gentamicin-loaded hydrogel in both groups. Lines represent median values.

Serum gentamicin concentrations peaked in both models at the first time point samples were taken after the surgery. The average concentration of serum gentamicin in the prophylaxis model at 3h postoperative was 613 ± 424 ng/mL, in the one-stage model at 1d postoperative it was 100.40 ± 61.91 ng/mL. These results suggest a burst release of gentamicin from the hydrogel immediately after local application in the intramedullary canal of the tibia, thereafter most gentamicin is released as the concentrations were below detection limit in most samples after 1 day (**Figure 3**). This burst release was observed in more detail as measured in the ECF locally.

The serum amoxicillin concentrations were variable between the sheep, but average concentrations do not show any peaks or throughs between day 1 and day 4 (**Figure 3**).

## Discussion

Local administration of antibiotics is increasingly being recognised as a means to improve success rates in managing ODRI. Current limitations to the application of local antibiotics to a wider range of potential patients include the absence of a versatile delivery method that will allow antibiotics to be administered during both primary and revision surgeries; to both prevent and treat infection; and that can be applied regardless of anatomical location or involved device. The injectable gentamicin-loaded hydrogel evaluated in the current study has shown efficacy in both the prevention and treatment of MSSA ODRI in a large preclinical model. The broad range of applications possible for such a biodegradable, injectable material is further enhanced by the markedly higher antibiotic concentrations at the surgical site in the post-operative period compared with conventional options such as ALBC.

In relation to the current evidence for local antibiotics, the addition of antibiotics to bone cement has been demonstrated to reduce the rates of both infection and aseptic loosening (Engesaeter et al., 2003). Furthermore, these findings are supported by a meta-analysis that demonstrated an 11% risk reduction for infection with local prophylactic antibiotics in high-risk trauma patients (Morgenstern et al., 2018). As ALBC is not resorbable, its utility for prophylaxis may be limited to cases that require multiple surgeries so the bone cement can be applied and removed during the following surgery. While the use of bone cement may be clinically indicated for reasons such as soft tissue defect management, its need for removal surgery is a limitation that precludes more widespread use. An approach to avoid this problem is through the off-label application of free antibiotic without a carrier into the surgical site. Vancomycin is commonly used as it comes in powder form, and has been demonstrated in a meta-analysis to reduce the rate of infection following spinal surgery (odds ratio 0.43) (Bakhsheshian et al., 2015). While application without a carrier can be useful for a broad patient population, the antibiotic choice is limited to those in powder form, may not have the appropriate antimicrobial coverage of infecting organisms, uncontrolled release may not suit the ideal pharmacodynamic properties of the antibiotic, and the lack of rigorous preclinical and clinical prospective trials means that the off-label use is largely guided by retrospective studies. We believe the development of a standardised carrier for local antibiotics has the potential to harness the benefits of local therapy while addressing the inherent limitations of current clinical practice. In the prophylaxis context, this gentamicin-loaded hydrogel has demonstrated superiority over intravenous perioperative antibiotic prophylaxis alone, which is the currently accepted gold standard for preventing ODRI (Classen et al., 1992; Foster et al., 2020). The total dose of antibiotic given in this prophylaxis setting is also a low risk, due to the low systemic concentrations achieved, and so the majority of active agent remains at the site it is most needed, rather than circulating through the bloodstream.

Treating established infection is a significantly different proposition to prevention. Treatment may involve surgical revision, long term antibiotic therapy and biofilm formation. The burden to the patient can be quite significant, and in order to minimise this burden there is increasing trend towards single stage revision surgery in appropriately selected patients (Gehrke et al., 2013). In single stage revision surgery, the use of ALBC is limited, much like in prevention, to those cases that would anyway have been using PMMA. Antibiotic hydrogels also offer many obvious advantages therefore over ALBC in treating established infection (Foster et al., 2020). Previous studies have shown a hydrogel combined with vancomycin and gentamicin applied in a single stage revision of ODRI due to MRSA showed positive results with four out of five cases leading to eradication of infection (Foster et al., 2020). Similarly, the same hydrogel when used in a two-stage revision protocol also demonstrated superiority over intravenous antibiotics for MRSA ODRI (Boot et al., 2021). By showing efficacy in a single stage exchange, the hydrogel may offer key local adjunctive antibiotics that may enable greater uptake of the single stage approach. The high local antibiotic concentration, without any need for removal surgery, or any lingering biomaterial, may make hydrogels a uniquely suitable mode of antibiotic delivery for treating established infection.

With regards to systemic toxicity, aminoglycosides are small and highly hydrophilic molecules, which causes any unbound drug to rapidly elute from the carrier material (Krause et al., 2016). This may cause systemic exposure with potential for secondary organ toxicity, as already described for collagen and calcium sulphate (Swieringa and Tulp, 2005; Wahl et al., 2011). While the gentamicin concentrations were high locally, the systemic exposure measured with serum sampling was low, with a maximum level of 1.0µg/mL 3 hours after surgery and undetectable by 2 days, consistent with the previous evaluation of a gentamicin/vancomycin hydrogel (Foster et al., 2020). The concentration of ECF release of gentamicin is clinically relevant as it exceeds the minimum inhibitory concentration (<1µg/mL) for *S. aureus* isolates from a large sample of infections worldwide (Jones et al., 2017), although of course how this relates to bacteria growing within tissues as microcolonies is not entirely clear. The toxicity and risk of associated adverse effects is likely to be minimal given Federal Drug Administration guidelines suggest that peak/trough serum levels should not exceed 12.0/2.0µg/mL with intravenous dosing (FDA, 2013).

The antibacterial effects of gentamicin may at least partially be explained by direct damage to the cell wall caused by gentamicin (Kadurugamuwa et al., 1993; Krause et al., 2016), which could also occur against biofilm-residing bacteria. The main antibacterial effect of aminoglycosides is through inhibition of protein synthesis (Krause et al., 2016) and activity against biofilm may be limited. However, the obvious positive effects of the gentamicin loaded hydrogel, coupled to the longstanding use of gentamicin in many local antibiotic carriers, supports its clinical application. Alternative antibiotics, with alternative mechanisms of action and pharmacodynamics, may potentially be superior, though this has not been shown in the literature to date.

Surprisingly, the amoxicillin concentrations measured in the extracellular fluid were found to be low and increased around day 6, which seems to contradict the steady state concentrations measured in the serum. A possible explanation may be that the probes from the ultrafiltration device are covered by gentamicin-loaded hydrogel, which may have blocked the systemically administered amoxicillin in the first few days.

Future directions to improve the hydrogel may be to broaden the spectrum of application of the hydrogel to Gram negative pathogens, and to achieve synergy (Watanakunakorn and Tisone, 1982). In addition, the hydrogel may be suitable for loading with novel antimicrobials, including bacteriophage and phage-derived enzymes such as peptidoglycan hydrolases (Onsea et al., 2020; Rotman et al., 2020; Sumrall et al., 2021). Increasing the spectrum of targeted pathogens is important to given low virulence infections due to Coagulase-negative Staphylococci and *Cutibacterium acnes* are increasing being recognised as causes of prosthesis loosening, non-union and post-operative pain (Foster et al., 2021).

In conclusion, this preclinical study demonstrates that locally delivered gentamicin by a hydrogel as carrier was highly effective at both preventing and treating MSSA ODRI without any concerning toxicity. The flexibility of application, high local antibiotic concentrations, and utility for both prevention and treatment identify it as a suitable candidate for further preclinical and translational studies.

## Data Availability Statement

The raw data supporting the conclusions of this article will be made available by the authors, without undue reservation.

## Ethics Statement

The animal study was reviewed and approved by the Ethical Committee of the Canton of Grisons in Switzerland.

## Author Contributions

WB: methodology, formal analysis, investigation, data curation, writing - original draft, writing - review & editing, and visualization. AF: writing - original draft and writing - review & editing. OG: investigation. DE: conceptualization and supervision. TS: investigation and methodology. MD: conceptualization, methodology, review and editing. SZ: conceptualization, methodology, and supervision. GR: conceptualization and supervision. TM: conceptualization, methodology, formal analysis, investigation, writing - review & editing, visualization, supervision, and project administration. All authors contributed to the article and approved the submitted version.

## Funding

This work was funded by AOTrauma as part for the clinical priority program on Bone Infection.

## Conflict of Interest

The authors declare that the research was conducted in the absence of any commercial or financial relationships that could be construed as a potential conflict of interest.

## Publisher’s Note

All claims expressed in this article are solely those of the authors and do not necessarily represent those of their affiliated organizations, or those of the publisher, the editors and the reviewers. Any product that may be evaluated in this article, or claim that may be made by its manufacturer, is not guaranteed or endorsed by the publisher.
